# Amyloid PET imaging and dementias: potential applications in detecting and quantifying early white matter damage

**DOI:** 10.1186/s13195-021-00933-1

**Published:** 2022-02-12

**Authors:** Anna M. Pietroboni, Annalisa Colombi, Tiziana Carandini, Luca Sacchi, Chiara Fenoglio, Giorgio Marotta, Andrea Arighi, Milena A. De Riz, Giorgio G. Fumagalli, Massimo Castellani, Marco Bozzali, Elio Scarpini, Daniela Galimberti

**Affiliations:** 1 Fondazione IRCCS Ca’ Granda Ospedale Maggiore Policlinico, Via F. Sforza 35, 20122 Milan, Italy; 2 University of Milan, Milan, Italy; 3 Dino Ferrari Center, Milan, Italy; 4 ‘Rita Levi Montalcini’ Department of Neuroscience, University of Torino, Turin, Italy; 5 Department of Neuroscience, Brighton and Sussex Medical School, University of Sussex, Brighton, UK

**Keywords:** amy-PET, Amyloid, Alzheimer’s disease, Non-AD dementias, White matter

## Abstract

**Purpose:**

Positron emission tomography (PET) with amyloid tracers (amy-PET) allows the quantification of pathological amyloid deposition in the brain tissues, including the white matter (WM). Here, we evaluate amy-PET uptake in WM lesions (WML) and in the normal-appearing WM (NAWM) of patients with Alzheimer’s disease (AD) and non-AD type of dementia.

**Methods:**

Thirty-three cognitively impaired subjects underwent brain magnetic resonance imaging (MRI), Aβ_1-42_ (Aβ) determination in the cerebrospinal fluid (CSF) and amy-PET. Twenty-three patients exhibiting concordant results in both CSF analysis and amy-PET for cortical amyloid deposition were recruited and divided into two groups, amyloid positive (A+) and negative (A−). WML quantification and brain volumes’ segmentation were performed. Standardized uptake values ratios (SUVR) were calculated in the grey matter (GM), NAWM and WML on amy-PET coregistered to MRI images.

**Results:**

A+ compared to A− showed a higher WML load (*p* = 0.049) alongside higher SUVR in all brain tissues (*p* < 0.01). No correlations between CSF Aβ levels and WML and NAWM SUVR were found in A+, while, in A−, CSF Aβ levels were directly correlated to NAWM SUVR (*p* = 0.04). CSF Aβ concentration was the only predictor of NAWM SUVR (adj *R*^2^ = 0.91; *p* = 0.04) in A−. In A+ but not in A− direct correlations were identified between WM and GM SUVR (*p* < 0.01).

**Conclusions:**

Our data provide evidence on the role of amy-PET in the assessment of microstructural WM injury in non-AD dementia, whereas amy-PET seems less suitable to assess WM damage in AD patients due to a plausible amyloid accrual therein.

## Introduction

Alzheimer’s disease (AD) is the most common neurodegenerative disorder and the main cause of dementia [1]. The hallmarks of AD pathology are the cortical deposition of beta-amyloid (Aβ) and the aggregation of tau protein into neurofibrillary tangles [2]. In addition to grey matter (GM) pathology, white matter (WM) changes were recently recognized as an important pathological feature of AD [3–6]. In particular, some studies demonstrated a higher WM lesion load (LL) in patients with cognitive decline showing pathological cerebrospinal fluid (CSF) levels of Aβ than those who were diagnosed as non-AD based on normal CSF Aβ concentration [5, 7]. Cerebral amyloid angiopathy (CAA) has also been shown to be a risk factor for accumulation of WM hyperintensities, with a strict association between amyloid burden in CAA patients [8]. However, the pathological substrate of WM damage in AD brains still remains unclear: the main hypothesis considers these WM changes as due to chronic ischaemic injury caused by cerebral microangiopathy [9, 10], while neuropathological studies show evidence of demyelination and axonal loss [11, 12]. Thus, other mechanisms could be implicated, including blood-brain barrier leakage, inflammation, neurodegeneration and CAA [11].

Positron emission tomography (PET) Aβ tracers (amy-PET) retention in the cerebral cortex is traditionally used to distinguish between AD and non-AD forms of dementia [13]. Interestingly, amy-PET retention has been more recently repurposed as an imaging marker for quantification of myelin loss and repair [14], particularly in the WM of patients with multiple sclerosis (MS) [15–17]. Amyloid tracers bind extensively to WM tissue and its uptake decreases with demyelination [15]. The application of amyloid tracers to the assessment of WM damage is justified by their strong affinity for myelin proteins as well as their solubility into myelin-associated lipid bilayer [15, 18]. Importantly, emerging evidence supports a direct connection between amyloid and myelin pathology [5, 17]. In light of these data, amy-PET may represent an intriguing tool to detect and quantify WM damage also when applied to neurodegenerative diseases.

To the best of our knowledge, there are only conflicting data in the literature on the relationship between measures of macro- and micro-structural WM damage and amy-PET uptake in WML and NAWM of AD and non-AD patients. Some studies found that WM amy-PET uptake is significantly higher in AD than in non-AD individuals [19], while other studies showed an equal uptake in AD and non-AD patients and/or in healthy controls [20]. Lastly, amy-PET uptake in WM seems to increase with age in both AD and non-AD patients and accumulates in WM areas whose anatomical distance from the GM makes it unlikely a GM spillover effect [21].

Against this background, aims of the current study were (1) to investigate amyloid tracer uptake in WML and NAWM of demented patients divided according to their amyloid positivity (A+ vs A−) and (2) to investigate possible correlations between amyloid tracer uptake, WMLL, CSF Aβ levels and brain volumes.

Based on the assumption that amy-PET is an imaging marker for quantification of myelin loss and repair, and on the assumption that WM damage represent a crucial feature in AD, we hypothesized a reduced tracer uptake in the WM in A+ patients.

## Materials and methods

### Subjects

Thirty-three patients with cognitive deficits were consecutively recruited at the Alzheimer Center of the University of Milan, Policlinico Hospital (Milan, Italy). All patients underwent a clinical interview, neurological and neuropsychological examination with Mini-Mental state examination (MMSE) assessment, and routine blood tests as routine diagnostic work up. Brain MRI, lumbar puncture (LP, for quantification of the CSF biomarkers Aβ) and amyloid-PET imaging were also performed within six months from the first clinical evaluation. Twenty-four patients were eventually diagnosed with AD, as confirmed by their pathological CSF Aβ levels, according to the criteria of the International Working Group guidelines [22]. Nine patients (all showing normal CSF Aβ levels) were diagnosed with a non-AD form of neurodegenerative dementia (specifically, frontotemporal dementia).

For CSF analysis, cut-off threshold of normality for Aβ_1-42_ was ≥ 600 pg/mL; a technique-related variability of ± 10% in determining Aβ levels was considered [23].

To minimize the risk of confounding variables associated with vascular comorbidities (i.e. subcortical vascular dementia, VaD) for the current study, we considered as suffering from a neurodegenerative form of dementia only those patients with an Hachinski Ischaemic Score (HIS) < 3, a periventricular and deep WM Fazekas score ≤ 2 and with no relevant history or risk factors for cardiovascular disease [5].

**Fig. 1:**
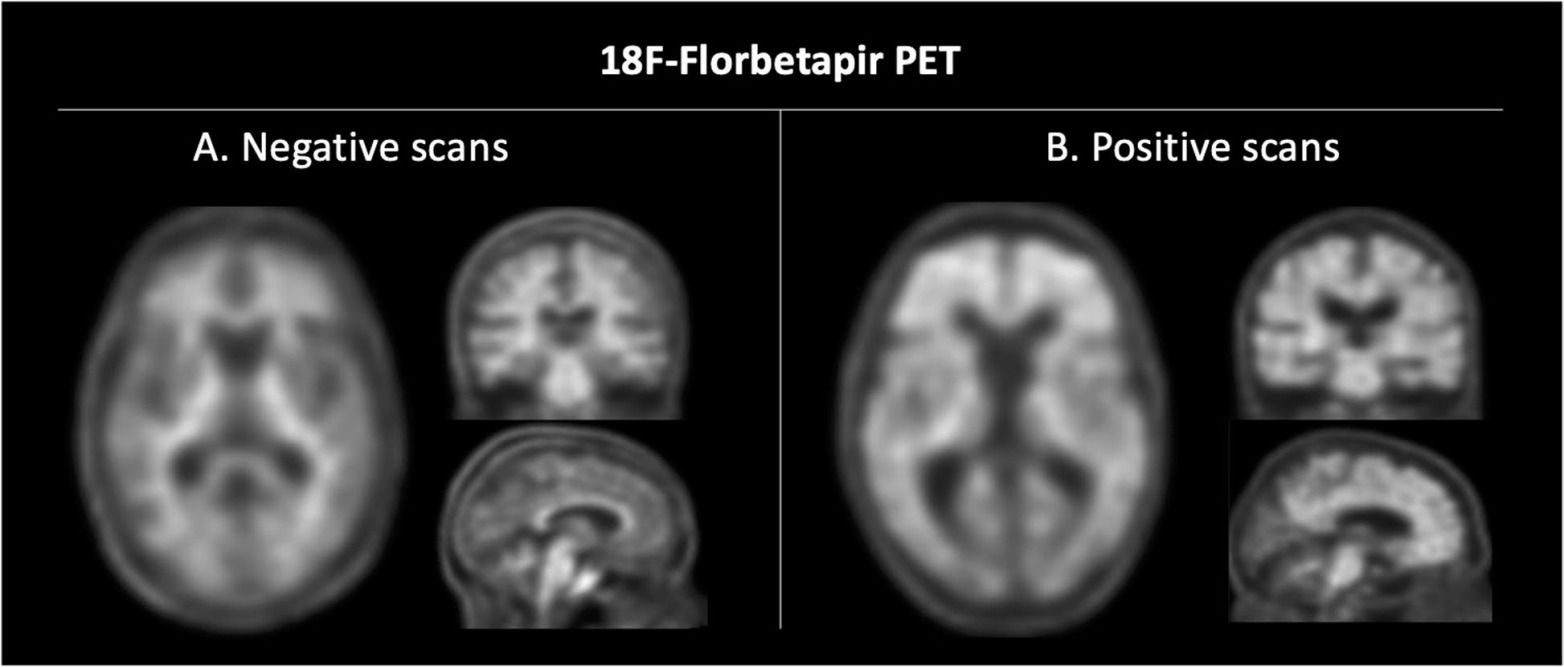
**A** Negative 18F-Florbetapir amyloid-PET scans, not showing amyloid plaque deposition: axial (left), coronal (right above), sagittal (right below). **B** Positive 18F-Florbetapir amyloid-PET scans, showing amyloid plaque deposition: axial (left), coronal (right above), sagittal (right below)

Consistent findings on CSF and amyloid PET imaging were also considered necessary for patient classification. For the purpose of this study, twenty-three patients only were retained according to their positivity on both CSF and amyloid PET imaging biomarkers. In detail, seventeen patients showing evidence of amyloid deposition as confirmed by both pathological CSF Aβ levels and positive amy-PET imaging were classified as A+ (all with a clinical diagnosis of AD), while six patients with CSF Aβ_1-42_ levels within the normal range and negative amy-PET were classified as A− (Fig. 1) (no one with a clinical diagnosis of AD).

The current study was approved by the institutional review board of Fondazione IRCCS Ca’ Granda Ospedale Maggiore Policlinico (Milan, Italy). All patients (or their legal guardians) and controls gave their written informed consent for this research before entering the study.

### CSF collection and Aβ determination

CSF samples were collected by LP performed in the L3/L4 or L4/L5 interspace. Following LP, CSF samples were centrifuged in 8000 rpm for 10 min. The supernatants were aliquoted in polypropylene tubes and stored at − 80 °C until use. CSF cell counts, glucose and proteins were determined. CSF Aβ_1-42_ was measured by using a commercially available sandwich enzyme-linked immunosorbent assay (ELISA) kit (Fujirebio, Ghent, Belgium).

### MRI and PET acquisition

All patients underwent an MRI examination using a 3T system (Philips Achieva, Eindhoven, The Netherlands). The acquisition protocol included the following: (1) a three-dimensional (3D) T1-weighted scan (relaxation time (TR) 9.90 ms; echo time (TE) 4.61 ms; flip angle 8°; slices thickness 1mm; gap 0); (2) a T2-weighted scan (TR 2492 ms; TE 78 ms; flip angle 90°; slices thickness 4 mm; gap 0) and (3) a fluid attenuated inversion recovery (FLAIR) scan (TR 11 000 ms; TE 125 ms; flip angle 90°; slices thickness 2 mm; gap 0).

PET scans were obtained with a Biograph TruePoint 64 PET/CT scanner (Siemens, Erlangen, Germany). All patients underwent 18F-florbetapir PET scanning at rest after intravenous injection of 370 MBq. Patients were positioned comfortably in a quiet room for at least 50 min. Each acquisition included a CT transmission scan of the head (55 mAs lasting 10 s) followed by a 20-min PET list-mode acquisition. PET sections were reconstructed with four 5-min frames to verify the absence of patient movement during the acquisition, and then with one frame of all 20 min in the form of transaxial images of 168 × 168 pixels (2 mm), using the iterative 3D TrueX algorithm with eight iterations and 14 subsets, with a Gaussian filter with full width at half maximum of 4 mm, and corrected for scatter and for attenuation using density coefficients derived from the low-dose CT scan of the head obtained with the same scanner, using the proprietary software.

### Neuroimaging data analysis

#### MRI analysis

All 3D T1-weighted scans were first visually inspected to exclude the presence of macroscopic artefacts. To quantify the macroscopic WM lesion load, lesions were segmented using the lesion growth algorithm as implemented in the Lesion Segmentation Tool (LST) toolbox version 2.0.15 (www.statistical-modelling.de/lst.html) for SPM12. Briefly, the algorithm first segments the T1 images into the main tissue classes and then calculates lesion belief maps onto the co-registered FLAIR images. By thresholding these maps with a threshold K value of 0.2 (determined by visual inspection of the results for the patients), an initial binary lesion map is obtained, and the region of interest (ROI) for the WM lesions is created. For each dataset, the WML was calculated, visually inspected to exclude the presence of macroscopic artefacts, and used for correlation analyses.

Lesions in T1-weighted images were filled using the lesion-filling tool in the LST toolbox. To obtain brain volumetrics, brain segmentation was performed using SPM12. The lesion-filled T1-series images were segmented according to GM, WM, and CSF tissue probability maps to generate the normalization deformation field into the Montreal Neurological Institute (MNI). Normal appearing WM (NAWM) maps were obtained by subtracting, for each subject, the WM lesion map from WM tissue after normalization to the MNI space. Finally, for each scan, we derived the GM, NAWM, and WML fractions, calculated as the ratio between each volume and total intracranial volume (TIV). Data were subsequently converted to percentages.

#### PET imaging analysis

The processing of PET imaging was performed using statistical parametric mapping software (SPM12, Wellcome Centre for Human Neuroimaging, University College London, UK). Using the ImCalc function of SPM, standardized uptake value (SUV) PET maps were derived as SUV = AC/(radiotracer dose/BW), where AC represents the activity concentration in a given voxel (kBq/ml), the radiotracer dose is the injected florbetapir dose corrected for residual activity in the syringe (MBq) and BW is body weight (kg).

SUV-PET images were co-registered to individual’s lesion-filled volumetric T1-weighted images. Using the NAWM, WML and GM segmentation maps previously obtained, we extracted the mean SUV for each patient’s tissue from the co-registered SUV-PET images.

To determine the evidence of amyloid cortical deposition (A+ vs A−) according to this technique, amy-PET data were both qualitatively and quantitatively analysed. Amy-PET data were first qualitatively analysed by a trained physiologist using a binary method of interpretation for relating “positive” or “negative” scans to neuropathologically defined categories of Aβ plaque density. This classification was further confirmed by comparing the GM mean retention of six previously defined cortical areas (anterior cingulate gyrus, orbital part of frontal lobe, superior parietal lobule, posterior cingulate gyrus, precuneus and temporal lobe) to the whole cerebellum, using a validated threshold for amy-PET SUV relative ratio (1.11) [24, 25].

### Statistical analysis

All statistical analyses were performed using Stata (v 14.0 MP) and SPM12. Due to the non-normal distribution of data (as preliminarily assessed by the Shapiro-Wilk test), all between-group comparisons were tested by nonparametric inferential statistical analyses (Mann–Whitney *U* test and Wilcoxon test for paired *t* tests).

All correlation analyses were performed using the Spearman correlation coefficient.

Multiple regression analyses were performed within each group with NAWM-SUVR as dependent variable and CSF Aβ levels as explanatory variable. Each regression model was adjusted to control for the potential effects of age, gender and WML.

The threshold of statistical significance was set to *p* < 0.05.

## Results

### Clinical and MRI data

The main demographic and MRI features of the recruited cohort of patients are summarized in Table 1. Of note, no differences in vascular risk factors (assessed by HIS) and the level of global cognition (assessed by the MMSE score) were found between A+ and A− subgroups.

**Table 1:** Clinical and MRI characteristics of cognitively impaired patients according to the evidence of Amyloid-beta accrual within the CNS (A+ vs A−) as provided by amyloid PET and CSF Ab levels

Demographic and conventional MRI variables	Subgroups		***A***− ***vs A+(p)***
	A− (***n*** = 6) Mean ± SD	A+ (***n*** = 17) Mean ± SD	
**Age, years**	77.6 ± 3.8	75.3 ± 7.8	*0.81*
**Gender, (F/M)**	2/4	7/10	*0.68*
**MMSE, raw score**	21.8 ± 3.3	23.3 ± 4.6	*0.57*
**Hachinski Ischaemic Score**	1.6 ± 1.2	0.7 ± 0.6	*0.09*
**CSF Ab levels, (pg/mL)**	986 ± 384	530 ± 91	***< 0.001***
**WML volume, % of TIV**	0.15 ± 0.14	0.40 ± 0.32	***0.049***
**NAWM volume, % of TIV**	24.47 ± 1.92	24.95 ± 1.72	*0.48*
**GMF volume, % of TIV**	35.96 ± 4.15	36.68 ± 2.57	*0.77*

Regarding conventional MRI analysis, A+ patients showed significantly higher WML than A− (*p* = 0.049). No differences in GM and NAWM volumes were found between the two subgroups (*p* = 0.77 and *p* = 0.48, respectively).

### SUVR is ubiquitously higher in A+ patients

Considering the whole study cohort, WML showed a lower SUVR when compared to NAWM (*p* < 0.001). No correlations between each tissue’s volume and SUVR were found in the GM, NAWM and WML.

**Fig. 2:**
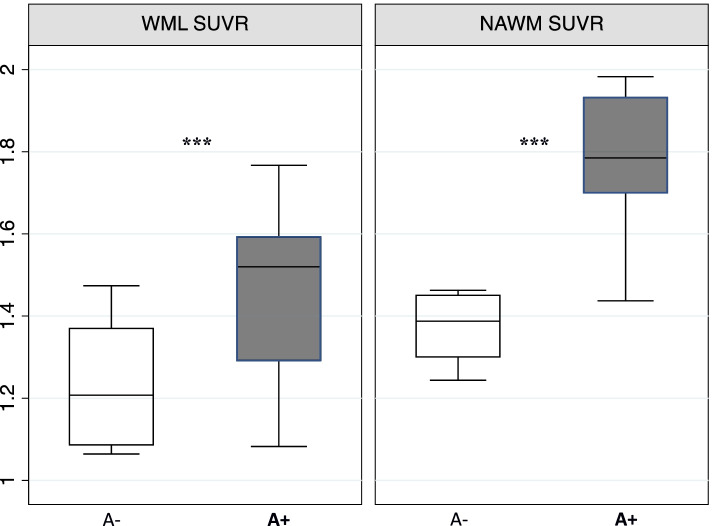
Boxplot showing SUVR distribution in WML and NAWM according to the amyloid-defined subgroups (A− vs A+). SUVR, standardized uptake volume ratio; WML, white matter lesions; NAWM, normal-appearing white matter; ***p < 0.001

When comparing amyloid tracer retention in the NAWM and WML across the amyloid-defined subgroups, A+ patients exhibited a significantly higher SUVR in both tissues (NAWM and WML) when compared to A− patients (*p* < 0.01 and *p* = 0.002, respectively; Fig. 2). As expected, GM SUVR was also higher in the A+ subgroup (*p* < 0.001).

A more extensive description of SUVR values of the study cohort is reported in Table 2.

**Table 2:** Amyloid tracer retention expressed as SUVR in WML, NAWM and GM; data are reported for the whole cohort and then compared according the amyloid-defined subgroups (A− vs A+)

Amyloid PET tracer retention	Whole cohort (***n*** = 23) Mean ± SD	Subgroups		***A***− ***vs A+ (p)***
		A− (***n*** = 6) Mean ± SD	A+ (***n*** = 17) Mean ± SD	
**SUVR WML (%)**	1.39 ± 0.23	1.23 ± 0.16	1.46 ± 0.21	***< 0.001***
**SUVR NAWM (%)**	1.66 ± 0.22	1.39 ± 0.09	1.79 ± 0.15	***< 0.001***
**SUVR GM (%)**	1.39 ± 0.16	1.09 ± 0.09	1.53 ± 0.12	***< 0.001***

### CSF Aβ levels predict NAWM microstructural integrity in A− patients

**Fig. 3:**
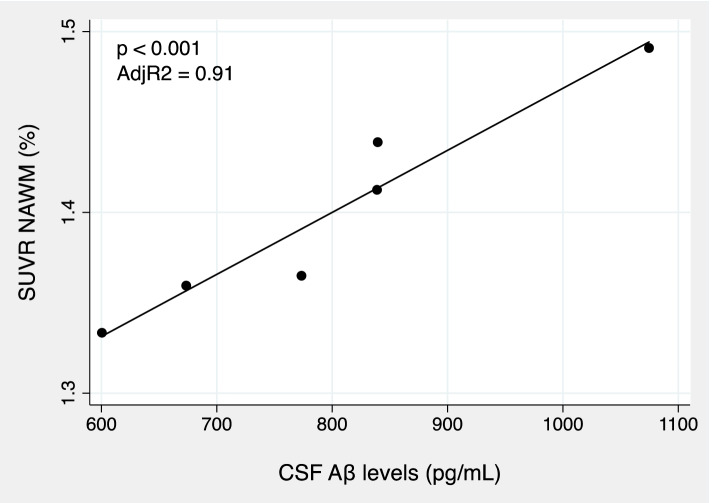
Scatter plot showing the linear regression of NAWM SUVR in function of CSF Ab levels in A− patients. CSF, cerebrospinal fluid; Ab, amyloid 1-42; NAWM, normal appearing white matter

Considering A− patients in isolation, CSF Aβ levels showed a strong positive correlation with NAWM SUVR (*ρ* = 0.45; *p* = 0.002). Multiple regression analysis revealed CSF Aβ concentration as the only predictor of amyloid tracer retention in NAWM (adj *R*^2^ = 0.91; *p* = 0.04; Fig. 3).

In A+ patients, no correlations between CSF Aβ levels and NAWM SUVR were found. Conversely, in this subgroup a robust positive correlation between NAWM and GM SUVR (ρ = 0.87; *p* < 0.001) was found.

## Discussion

In this study, we reported WM SUVR data of amy-PET in AD and non-AD demented patients with the aim to investigate possible differences between the two groups.

First, we found surprisingly that amyloid tracer uptake was higher in all brain tissues of A+ compared with A− patients, suggesting an aberrant retention of Aβ tracers in AD brains involving not only the cortex but also the WM. This finding appears to be remarkable considering that amyloid tracer uptake has been shown to decrease with an increasing accumulation of WM damage [15, 17, 18]. This is even more remarkable considering that our A+ patients showed higher WML loads than A− patients [5]. Given the lack of unequivocal interpretation of this finding, a range of hypotheses may be explored to account for this data. The simplest explanation might be the spillover of cortical signal into subcortical areas of WM close to GM tissue. Another explanation might be a more remarkable deposition of Aβ in the WM of A+ brains. Finally, interference of Aβ peptide deposits in the walls of small to medium size blood vessels might account for this increased retention of amyloid tracer in A+ patients’ WM, similarly to that observed in patients with cerebral amyloid angiopathy (CAA). As explained below, important for this speculation is also our finding of increased Aβ deposition in the NAWM of A+ patients. However, considering the relatively small sample size of this study, further investigations are necessary to confirm these results and address their interpretation.

Second, we did not identify any association between CSF Aβ levels and either WML or NAWM SUVR in A+ patients, while, in A− patients, CSF Aβ levels were directly correlated with NAWM SUVR. Furthermore, CSF Aβ concentration was the only predictor of NAWM SUVR in A−. This finding confirms the predictive role of Aβ on myelin damage, appearing in line with previous studies, which described a common pattern within different pathological conditions: the lower the uptake, the lower the CSF Aβ concentration [17].

The most interesting finding of our study is that the tracer uptake in the NAWM is not univocal, but it follows a different trajectory in A+ and A− patients. Based on preliminary pathological studies, which revealed the presence of Aβ deposits in the WM of A+ patients [26] alongside increased levels of soluble Aβ peptide [27], we hypothesize that the greater amyloid tracer uptake we observed in A+ patients might depend on Aβ deposition in the WM tissue. Although most of Aβ plaques in the WM were located immediately beneath the GM tissue, there were some Aβ deposits in the deep region of the WM that cannot be merely explained by spillover of GM signal [26]. This is consistent with post-mortem data published by Rutten-Jacobs and colleagues, who demonstrated the presence of Aβ plaques in periventricular WM areas of brains from nondemented individuals [3, 28]. These observations indicate that Aβ deposition in the WM tissue contributes to AD pathology. Although amy-PET seems less suitable for the clinical assessment of WM damage in AD patients, because of a plausible amyloid accrual therein, it seems an extremely promising tool for the detection of microstructural WM injury in non-AD brains, as well in other neurological diseases.

### Limitations

There are some limitations when considering our study. First, we acknowledge that this is an exploratory investigation that requires confirmation in future studies on larger cohorts of patients. Admittedly, the sample size included here was limited by the conservative decision to consider only patients exhibiting concordant results between CSF analysis and amy-PET for cortical amyloid deposition. Moreover, the data interpretation of Aβ deposition in the WM tissue of A+ patients is based on assumptions that need confirmation through advanced imaging techniques or neuropathology. Lastly, we know that partial volume effect is an unavoidable issue on quantification accuracy when dealing with brain PET imaging, mainly due to the limited spatial resolution. Some previous studies have used partial volume correction (PVC) for white matter SUVR quantification. However, quantitative amy-PET imaging is usually conducted without PVC, due to the lack of a standardized and widely accepted PVC method, and some authors reported worse results and comparability using PVC as compared to native images. We applied an iterative spatial resolution reconstruction algorithm (TrueX) to images before performing SUVR quantification. Although TrueX cannot be fully equated to a PVC method, it already reduces significantly the partial volume effect.

## Conclusions

This study provides evidence on the role of amy-PET in the assessment of microstructural WM injury in non-AD dementia, whereas amy-PET seems less suitable to assess WM damage in AD patients due to a plausible amyloid accrual therein. Therefore, a specific study on AD patients is worth to be specifically performed. A replication in a larger cohort of patients is required to confirm these preliminary data.

## Data Availability

The datasets used in this study are available from the corresponding author upon reasonable request.
